# The potassium channel KCa3.1 constitutes a pharmacological target for astrogliosis associated with ischemia stroke

**DOI:** 10.1186/s12974-017-0973-8

**Published:** 2017-10-16

**Authors:** Mengni Yi, Tianjiao Wei, Yanxia Wang, Qin Lu, Gaoxian Chen, Xiaoling Gao, Herbert M. Geller, Hongzhuan Chen, Zhihua Yu

**Affiliations:** 1 Department of Pharmacology, Institute of Medical Sciences, Shanghai Jiao Tong University School of Medicine, Shanghai, 200025 China; 2 Experimental Teaching Center of Basic Medicine, School of Medicine, Shanghai Jiao Tong University, Shanghai, 200025 China; 3 Developmental Neurobiology Section, Division of Intramural Research, National Heart, Lung, and Blood Institute, National Institutes of Health, Bethesda, MD 20892 USA

**Keywords:** Astrocytes, Mouse, GFAP, Ischemia, Calcium

## Abstract

**Background:**

Reactive astrogliosis is one of the significantly pathological features in ischemic stroke accompanied with changes in gene expression, morphology, and proliferation. KCa3.1 was involved in TGF-β-induced astrogliosis in vitro and also contributed to astrogliosis-mediated neuroinflammation in neurodegeneration disease.

**Methods:**

Wild type mice and KCa3.1^−/−^ mice were subjected to permanent middle cerebral artery occlusion (pMCAO) to evaluate the infarct areas by 2,3,5-triphenyltetrazolium hydrochloride staining and neurological deficit. KCa3.1 channels expression and cell localization in the brain of pMCAO mice model were measured by immunoblotting and immunostaining. Glia activation and neuron loss was measured by immunostaining. DiBAC4 (3) and Fluo-4AM were used to measure membrane potential and cytosolic Ca^2+^ level in oxygen-glucose deprivation induced reactive astrocytes in vitro.

**Results:**

Immunohistochemistry on pMCAO mice infarcts showed strong upregulation of KCa3.1 immunoreactivity in reactive astrogliosis. KCa3.1^−/−^ mice exhibited significantly smaller infarct areas on pMCAO and improved neurological deficit. Both activated gliosis and neuronal loss were attenuated in KCa3.1^−/−^ pMCAO mice. In the primary cultured astrocytes, the expressions of KCa3.1 and TRPV4 were increased associated with upregulation of astrogliosis marker GFAP induced by oxygen-glucose deprivation. The activation of KCa3.1 hyperpolarized membrane potential and, by promoting the driving force for calcium, induced calcium entry through TRPV4, a cation channel of the transient receptor potential family. Double-labeled staining showed that KCa3.1 and TRPV4 channels co-localized in astrocytes. Blockade of KCa3.1 or TRPV4 inhibited the phenotype switch of reactive astrogliosis.

**Conclusions:**

Our data suggested that KCa3.1 inhibition might represent a promising therapeutic strategy for ischemia stroke.

**Electronic supplementary material:**

The online version of this article (10.1186/s12974-017-0973-8) contains supplementary material, which is available to authorized users.

## Background

During pathological brain insults such as cerebral ischemia, a rapid increase of intracellular calcium initiates apoptotic and necrotic cell death and reactive gliosis. Several lines of evidence suggest that [Ca^2+^]_i_ oscillations evoked by focal ischemia spread through the astrocytes and cause damage in distal regions of the central nervous system (CNS) [1]. KCa3.1, a Ca^2+^-activated potassium channel, is involved in regulating membrane potential during activation of non-excitable inflammatory and structural cells, including astrocytes, in response to Ca^2+^ influx [2–4]. As such, KCa3.1 is a promising target to ameliorate the phenotype switch of astrocytes and microglia from resting to astrogliosis and microglia activation in ischemia, traumatic brain injury, Alzheimer’s disease (AD) as well as spinal cord injury [5, 6].

Most recently, we reported that KCa3.1 was increased in reactive astrocytes as well as neurons in the brains of both mouse models of AD and AD patients. Furthermore, the blockade of KCa3.1 resulted in a decrease in astrogliosis and an attenuation of memory deficits in the AD mouse model [7]. After ischemic stroke in rats, blood-brain barrier endothelial cells exhibited KCa3.1 protein and activity, and pharmacological blockade of KCa3.1 significantly reduced Na^+^ uptake and cytotoxic edema [8]. Moreover, in a mouse model of ischemia, genetic deletion and wild type mice treated with the KCa3.1 blocker TRAM-34 resulted in a decrease in infarct areas and improved neurological deficits [9]. These findings suggest that KCa3.1 channels are important in the process of stroke, and that their blockade might prove useful as therapy in stroke with upregulated KCa3.1 expression.

Over the past few years, a large number of studies suggested that astroglial calcium influx after ischemia could be mediated by the activation of Ca^2+^ permeable cation channels such as transient receptor potential (TRP) channels. The transient receptor potential vanilloid 4 (TRPV4) channel is widely expressed in the CNS, with demonstrated function not only in neurons and astrocytes but also in endothelial cells of cerebral arteries [10]. During cerebral ischemia, TRPV4 was over-activated by cytotoxic edema or the metabolites of arachidonic acid (AA), and TRPV4-mediated Ca^2+^ entry likely played a role in the intracellular Ca^2+^ overload [11]. Blockade of the TRPV4 channel reduced the damage to hippocampal pyramidal neurons and astrocytes upon oxidative stress [12] or oxygen-glucose deprivation [13] in vitro and reduced brain infarction in a mouse model of focal cerebral ischemia [14]. At present, the signal transduction pathways used by TRPV4 to induce astrogliosis are not well defined but seem mostly related to calcium overload of the cells, as TRPV4 channels are involved in ischemia-induced calcium entry in reactive astrocytes and thus, might participate in the pathogenic mechanisms of astroglial reactivity following ischemic insult [15].

The present study aims to investigate the mechanisms by which KCa3.1 regulates Ca^2+^ entry via TRPV4 channels, leading to reactive astrogliosis during ischemia stroke. We show, for the first time, a potential co-localization between KCa3.1 and TRPV4 channels in astrocytes. This co-localization allows KCa3.1 to maintain a negative membrane potential during astrogliosis, thus increasing the driving membrane potential for Ca^2+^ influx through TRPV4 channels. These strongly suggest that KCa3.1 is involved in reactive astrogliosis in the process of stroke, making it a promising target for the development of novel therapies.

## Methods

### Permanent focal cerebral ischemia

The study (ethics protocol number: A-2015-010) was approved by the Animal Care and Use Committee of the Shanghai Jiao Tong University School of Medicine, Shanghai, China. KCa3.1^−/−^ mice were obtained from the Jackson Laboratory as described precisely [16–18]. 10–12 week old male C57BL/6 wild type mice and KCa3.1^−/−^ mice were housed in a specific pathogen-free animal facility. A permanent focal cerebral ischemia model was prepared in accordance with the guidelines as described previously [19]. In brief, adult male mice weighing 23 to 27 g were anesthetized with 2% chloral hydrate and body temperature was maintained at 37 ± 0.5 °C throughout the surgery using a heating pad and lamp (ALC-HTP, Shanghai Alcott Biotech Co. Ltd). To induce permanent focal cerebral ischemia, a silicon rubbed-counted suture with a tip diameter of 0.22 mm (L2000, AAA, Guangzhou Jialing Biiotech Co, Ltd.) was inserted into the left external carotid artery to block the middle cerebral artery. Transcranial laser Doppler (moorVMS-LDF2) was used to monitor cerebral blood flow (CBF) to assure reduction of CBF through the surgery (Additional file 1: Figure S1). The sham-operated mice experienced the same surgical operations except for the silicon rubbed-counted suture inserted.

### Stroke study population and quality control

The operators were not involved in data analysis and acquisition. The observers performed the surgeries, and parameters evaluation was unaware of the group to which each mouse belonged. The following conditions excluded mice from end-point analyses (exclusion criteria): (1) < 80% reduction in CBF; (2) subarachnoid hemorrhage or the brain parenchyma bleeding (as macroscopically assessed during brain sampling); (3) neurological score = 0 (6 h, 24 h after pMCAO); and (4) operation time > 10 min. In total, 158 mice (86 C57BL/6 WT, 72 KCa3.1^−/−^) were used in this study. Of the 120 mice subjected to pMCAO, 12 mice (10%) met at least one exclusion criterion after randomization and, therefore, were withdrawn from the study.

### Measurement of neurological deficits

Mice were studied for neurological deficits at 3, 6, and 24 h after pMCAO as described previously [20]. Briefly, neurological findings were scored on a 5-point scale: 0, no observable neurological deficits (normal); (1) failure to extend the right forepaw (mild); (2) circling to the contralateral side (moderate); (3) loss of walking or righting reflex (severe); (4) dead. The observers were unaware of the group to which each mouse belonged.

### Determination of infarct volume

Groups of mice were euthanized at 3, 6, and 24 h after MCAO. Brains were quickly removed and chilled at − 20 °C for 20 min to slightly harden the tissue. Then brains were sectioned into five 1 mm-thick coronal slices starting from the frontal pole. All sections were stained with 2% 2,3,5-triphenyltetrazolium hydrochloride in the dark for 20 min at 37°C and flipped every 5 min for staining of anterior and posterior faces [21]. Finally slices were fixed in 4% paraformaldehyde overnight at 4 °C. ImageJ was used to measure the infarct area of each brain.

### Immunohistochemistry

For immunofluorescence staining of serial brain coronal sections (12 μm) and cultured cells, the tissues and cells were blocked with 1% bovine serum albumin and 1% goat normal serum 1 h at room temperature. Sections and cells were incubated at 4 °C overnight with primary antibodies: mouse anti-KCa3.1 (1:100; Alomone Labs), rabbit anti-GFAP (1:500; Dako); rabbit anti-Iba1 (1:500; Abcam); rabbit anti-NeuN antibody (1:500; Millipore), rabbit anti-TRPV4 (1:200; Alomone Labs). The sections and cells were incubated with following secondary antibodies: Alexa Fluor 555 goat anti-rabbit IgG (1:500; Invitrogen), Alexa Fluor 488 goat anti-mouse IgG (1:500; Invitrogen) for 1 h at room temperature. Then washed with PBS and stained with DAPI (4′, 6-diamidino-2-phenylindole).

### Data collection and statistics of immunofluorescence

Twelve-micrometer-thick brain slices were collected from mice and four slices at 120 μm intervals from each brain were used to examine GFAP, Iba-1, and NeuN positive cells.

The average optical density of GFAP. At least three microscopic photographs of vision were selected in hippocampus or cortex of each immunofluorescence hemisphere slice. Leica·TCS·SP8 Laser scanning confocal microscope was used to capture photographs, which were obtained under the same confocal settings. The area of the AOI (area of interest, area) and the integral optical density (IOD) value was measured using the Image-Pro Plus 6.0 software as in previous study [22], and the IOD/area was calculated to obtain the mean IOD in each image. At last, the final mean IOD of each slice was determined by the average of the mean IOD in each image.

Cell counting. At least three fields were captured in each slice with the same reference position for quantification. The numbers of Iba1^+^ and NeuN^+^ cells per 1 mm^2^ in each slice were counted using the Image-Pro Plus 6.0 software. The data from at least three photographs of each hemisphere slice were averaged as one value and values from three slices were calculated.

Analysis of co-localization. For analysis of TRPV4 and KCa3.1 co-localization, images were processed and analyzed using Leica LAS AF Lite software (Leica, Germany). Pearson correlation was used to express the degree of co-localization as described early [23, 24]. The co-localization of KCa3.1 and TRPV4 in the overlap of the two channels was assessed using the co-localization tool in Leica LAS AF Lite software. The Pearson correlation values range from − 1 to + 1. A correlation of 1 indicates complete co-localization between the two proteins. A correlation of − 1 indicates a negative interaction, and a correlation of 0 indicates no co-localization between the two proteins [24].

### Primary culture of astrocytes

Primary astrocyte cultures were prepared from neonatal (0–2 days old) C57BL/6 wild type or KCa3.1^−/−^ mouse brains as described previously [17]. Briefly, the cerebral cortices were dissected out and dissociated into a single cell suspension. When cells grew to confluence (10-14 days later), flasks were shaken overnight (200 rpm, 37 °C) and the medium exchanged to remove adherent microglia and oligodendrocytes. The purified astrocytes were plated into plates in serum-containing DMEM. After once again reaching confluence, the medium was exchanged for serum-free DMEM for 24 h before treatments. In some cases, the cells were pretreated with the blockers 30 min before oxygen-glucose deprivation.

### Oxygen-glucose deprivation and drug exposure

Confluent astrocytes were grown in serum-free DMEM for 24 h before OGD, at which time the culture medium was replaced with glucose/glutamine-free DMEM medium after a gentle cell washing with the same medium. The serum-free and glucose/glutamine-free DMEM medium was balanced for 30 min with 95% (*v*/*v*) N_2_, 5% (*v/v*) CO_2_ at 37°C before OGD. Then cells were exposed to hypoxia for different time points in a small anaerobic chamber filled with 95% (*v/v*) N_2_ and 5% (*v/v*) CO_2_ at 37 °C. Drugs and inhibitors were pretreated to the cells 30 min before OGD. A Cell Counting Kit-8 (CCK-8, Dojindo Laboratories, Kumamoto, Japan) was used to measure cell viability [25].

### Western blotting

Mouse brain tissues or astrocytes were homogenized in RIPA buffer (25 mM Tris pH 7.4, 150 mM NaCl, 1% NP-40) containing 0.1% sodium dodecyl sulfate (SDS) and 4% protease inhibitor (complete protease inhibitor cocktail, Roche). Tissue lysates were centrifuged at 13500 rpm for 30 min at 4 °C, and supernatants were collected. Total lysates were diluted in 2 × SDS sample buffer (120 mM Tris/HCl, 10% SDS, 20% glycerine, 20% 2-mercaptoethanol, pH 6.8) to a final concentration of 2 μg/μl and were used for western blotting. The following primary antibodies were used: anti-KCa3.1 (1:500; Abcam), anti-TRPV4 (1:500; Alomone Labs), anti-GFAP (1:2000; Z0334, Dako), and anti-β-actin (1:1000; Santa Cruz). HRP-conjugated anti-rabbit or anti-mouse IgG secondary antibodies (1:3000; Amersham Biosciences) were used for 1 h at room temperature. ImageJ software was used to quantify the protein bands and normalized to the actin band, which served as loading control [26].

### Cell viability

Cell viability was assessed using a Cell Counting Kit-8 (CCK-8, Dojindo Molecular Technologies) as previously described [4]. Briefly, the confluent astrocytes were cultured in serum-free media for 24 h and were then treated with OGD for different time points (1, 3, 4 6, 12 h). Ten microliter CCK-8 was added to each well of the 96-well plate and then was placed in a CO_2_ incubator for 2 h. Measure the absorbance at 450 nm with a microplate reader.

### Membrane potential measurement

Bis-(1,3-dibutylbarbituric acid) trimethine oxonol [DiBAC4(3)], the potentiometric fluorescent dye, was used to measure membrane potential as described previously [27]. Briefly, primary cultured astrocytes were loaded with 100 nmol/l DiBAC4 (3) for 20 min to ensure dye distribution across the cell membrane at 37 °C in an incubator. 1-EBIO (200 μM) or RN1747 (10 μM) was added to the cells at 60 s. A TCS SP8 confocal laser-scanning microscope (Leica, Germany) was used to evaluate relative changes in membrane potential by monitoring DiBAC4(3) fluorescence. TRAM-34 (1 and 10 μM) or HC 067047 (10 μM) was added 1 h before the experiment. DiBAC4(3) fluorescence was measured at 530 nm with excitation at 488 nm. Confocal images were taken and stored every 1 s for 1800 s.

### [Ca^2+^]_i_ measurement

Cytosolic Ca^2+^ ([Ca^2+^]_i_) was measured as described previously [27]. Briefly, astrocytes were loaded with 5 μM Fluo-4 AM (MAIBIO, Shanghai, China) for 30 min at 37 °C in an incubator, rinsed, and incubated in DMEM with the appropriate test reagents. Baseline fluorescence was measured for the first 60 s, and then 1-EBIO (200 μM) or RN1747 (10 μM) was added to the cell plate. A TCS SP8 confocal laser-scanning microscope (Leica, Germany) was used to evaluate relative changes in intracellular calcium concentration ([Ca^2+^]_i_) by monitoring Fluo-4 fluorescence. TRAM-34 (1 and 10 μM) or HC 067047 (10 μM) was added 1 h before the experiment. Fluo-4 fluorescence was measured at 510 nm, with excitation at 488 nm. Confocal images were taken and stored every 1 s for 360 s.

### Statistical analysis

All data are presented as means ± SEM. Statistical analyses were performed using Prism software (GraphPad Software, Inc., La Jolla, CA, USA). Data were tested for Gaussian distribution with the Kolmogorov–Smirnov normality test and then analyzed by one-way ANOVA and Dunnett’s post hoc tests. Data were analyzed with unpaired, two-tailed Student’s *t* test when comparing between two groups, or the non-parametric Mann–Whitney test was applied. Statistical significance was set at *p* < 0.05.

## Results

### Upregulation of KCa3.1 expression in the brains of pMCAO mice

**Fig. 1:**
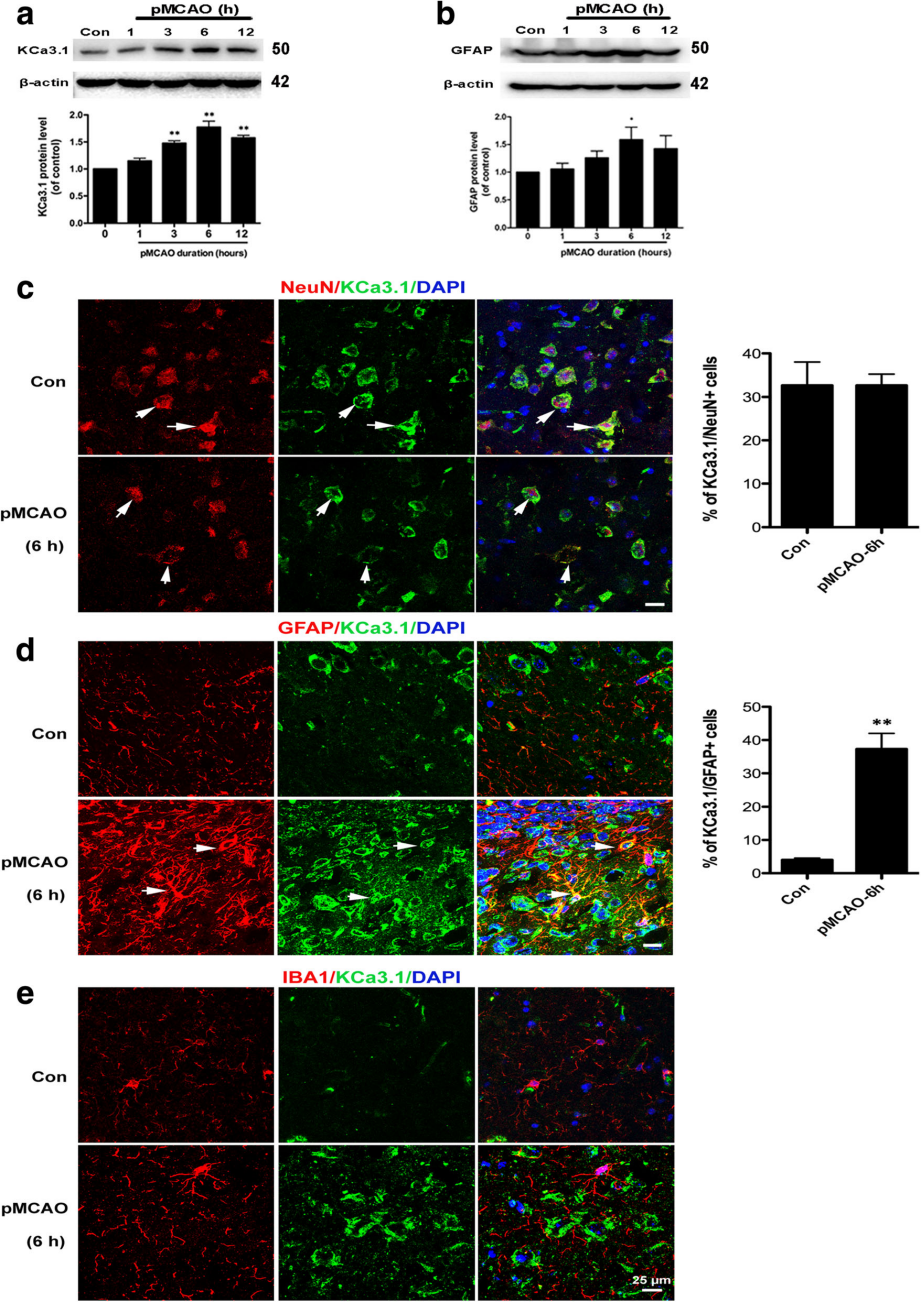
Upregulation of KCa3.1 channels and GFAP in mouse brains following pMCAO. **a**, **b** Western blot analysis of lysates from 10-week-old male WT mice following 1, 3, 6, or 12 h of pMCAO analyzed by antibodies to KCa3.1 (**a**) and GFAP (**b**). Data represent the means ± SEM of KCa3.1 and GFAP density normalized to β-actin values for *n* = 3. ^*^ *p* < 0.05, ^**^ *p* < 0.01, one-way ANOVA followed by the Dunnett’s multiple comparison test compared with control. Con control. **c–e** Cellular localization of KCa3.1 channels in mouse brains following pMCAO. Double immunofluorescence analysis of KCa3.1 (green) levels in (**c**) neurons (NeuN, red), (**d**) astrocytes (GFAP, red), and (**e**) microglia (Iba1, red) of control and 6 h pMCAO mouse brain. DAPI (blue) was used to label nuclei. Quantification of the percentage of NeuN^+^ (**c**) or GFAP^+^ (**d**) cells colabeled for KCa3.1 at 6 h pMCAO compared with the control group. ^**^ *p* < 0.01, unpaired, two-tailed Student’s *t* test compared with control mice (*n* = 4). Scale bar: 25 μm. Con control

Most recently, we reported KCa3.1 was increased in reactive astrocytes in the brains of both mouse models of AD and AD patients, and pharmacological inhibition or genetic deletion of KCa3.1 reduced astrogliosis-induced neuronal loss in the AD mouse model [7]. Based on this, we hypothesized that KCa3.1 is also mechanistically involved in astrogliosis-mediated neuronal damage following ischemic stroke. Here, we chose a mouse model of pMCAO as described previously [19]. Following MCAO blockage, the expression of both KCa3.1 and GFAP was significantly increased in the infarcted hemisphere of pMCAO mice as detected by western blotting (Fig. 1a, b) for up to 12 h (Additional file 2: Figure S2).

Co-immunostaining of KCa3.1 with specific for neurons, astrocytes, and microglia was performed on brain sections of control and 6 h pMCAO mice. KCa3.1 expression was found in NeuN^+^ neurons in the brains of both control and 6 h pMCAO mice (Fig. 1c). Less KCa3.1 expression was detected in the neurons of pMCAO mice because of the cells loss during the pathology process of ischemia compared to the control group, but there was no obviously difference in the percentage of the co-localization between pMCAO and control group. In control mice, a low level of expression of KCa3.1 was detected in GFAP^+^ astrocytes (Fig. 1d). However, at 6 h pMCAO, we detected a clear co-localization between KCa3.1 and GFAP^+^ reactive astrocytes in the infarct regions (*p* < 0.01, Fig. 1d), which is consistent with our previous reports in AD [7]. Because Chen et al. [9] reported acutely isolated microglia from the brains of transient MCAO mice with 8 days of reperfusion exhibit increased function of KCa3.1 channels, so we labeled sections from the 6 h pMCAO for KCa3.1 and the microglial marker Iba1. There was no obvious co-localization between KCa3.1 and microglia at 6 h pMCAO (Fig. 1e).

### Genetic KCa3.1 deletion reduces infarct area and improve neurological deficit in pMCAO

**Fig. 2:**
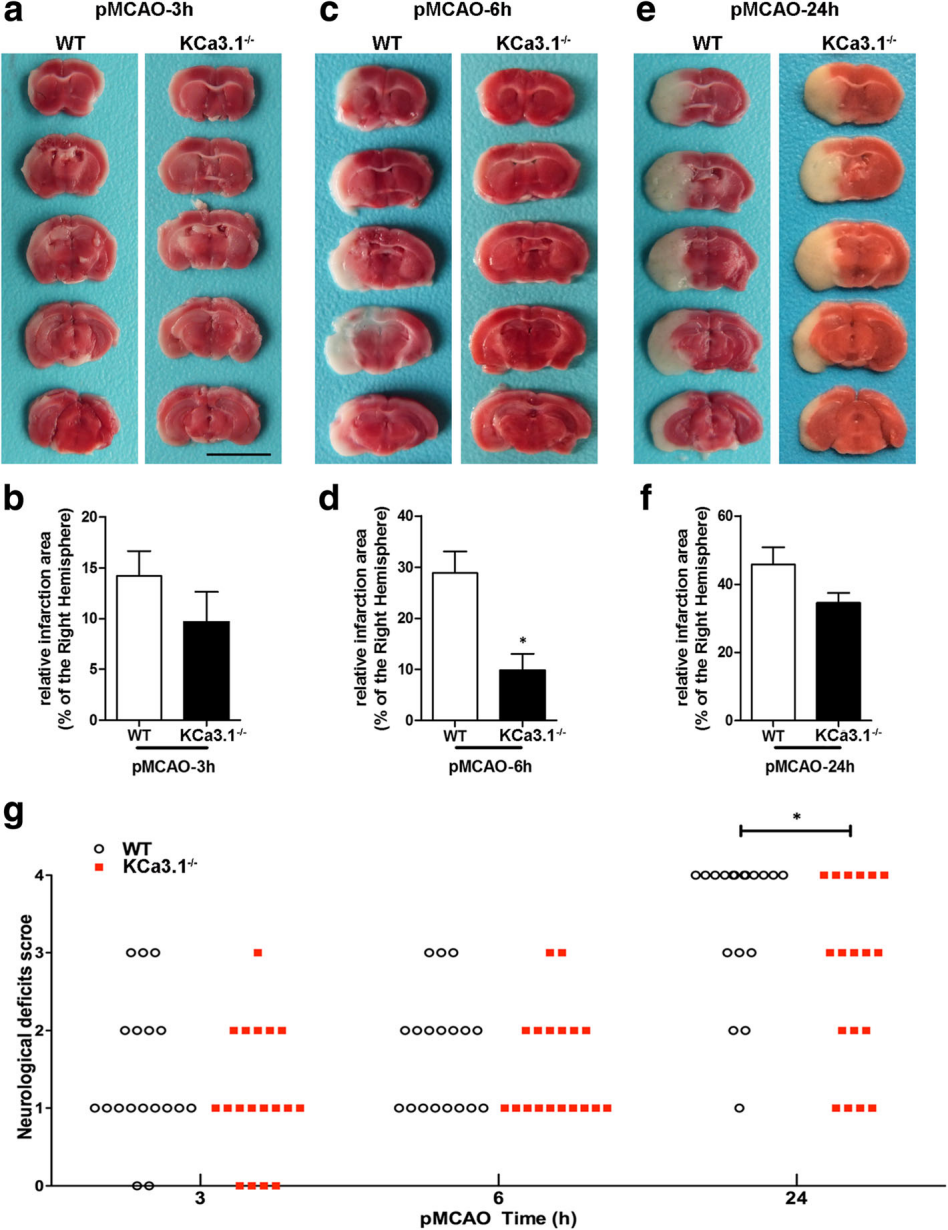
KCa3.1 deficiency reduces infarction volume and improves of neurological conditions. Focal cerebral ischemia was induced by pMCAO. **a**, **c**, and **e** Representative TTC staining of five corresponding coronal brain sections of a 10-week-old male WT mouse and a 10 week-old male KCa3.1^−/−^ mouse after 3 h (**a**), 6 h (**c**), and 24 h (**e**) of pMCAO. **b**, **d**, and **f**. Quantitative analysis of infarction volume in a, c, and e, respectively. Data are presented as means ± SEM. *n* = 6. ^*^ *p* < 0.05, unpaired, two-tailed Student’s *t* test compared with ischemic WT group. **g** Neurological deficits were assessed at 3, 6, and 24 h after pMCAO. Significance: ^*^ *p* < 0.05 vs WT group (*n* = 18). Mann–Whitney test compared with WT mice. The survival rate of 24 h after pMCAO was obviously higher in the KCa3.1^−/−^ group than that in the WT group (66.7 vs 37.5%, respectively), and significant differences was observed (*p* = 0.0486, chi-square test)

In order to study the role of KCa3.1 deficiency on the pathological processes of ischemic stroke, we subjected wild type (WT) and KCa3.1 gene deletion (KCa3.1^−/−^) mice to 3, 6, and 24 h of pMCAO and assessed infarct volumes and neurological deficits (Fig. 2). As shown in Fig. 2a–f, the infarct area measurements by TTC staining in pMCAO brains indicated that infarct volumes in KCa3.1^−/−^ mice was smaller than those in WT mice, and infarct areas in KCa3.1^−/−^ mice were significantly reduced by 19% at 6 h after pMCAO compared with those in WT mice (*p* < 0.05, Fig. 2c, d) but there was no significant difference in infarct measurement at 3 h (*p* = 0.3085, Fig. 2a, b) and 24 h after stroke (*p* = 0.0951, Fig. 2e, f). Furthermore, the neurological deficit scores of KCa3.1^−/−^ mice were significantly lower than those of WT mice at 24 h after pMCAO (*p* < 0.05, Fig. 2g). The survival rate of 24 h after pMCAO was significantly higher in KCa3.1^−/−^ mice than that in WT mice (*p* < 0.05, 66.7% (12/18) vs 37.5% (6/16), Fig. 2g).

### Genetic KCa3.1 deficiency reduces neuron loss and gliosis induced by pMCAO

**Fig. 3:**
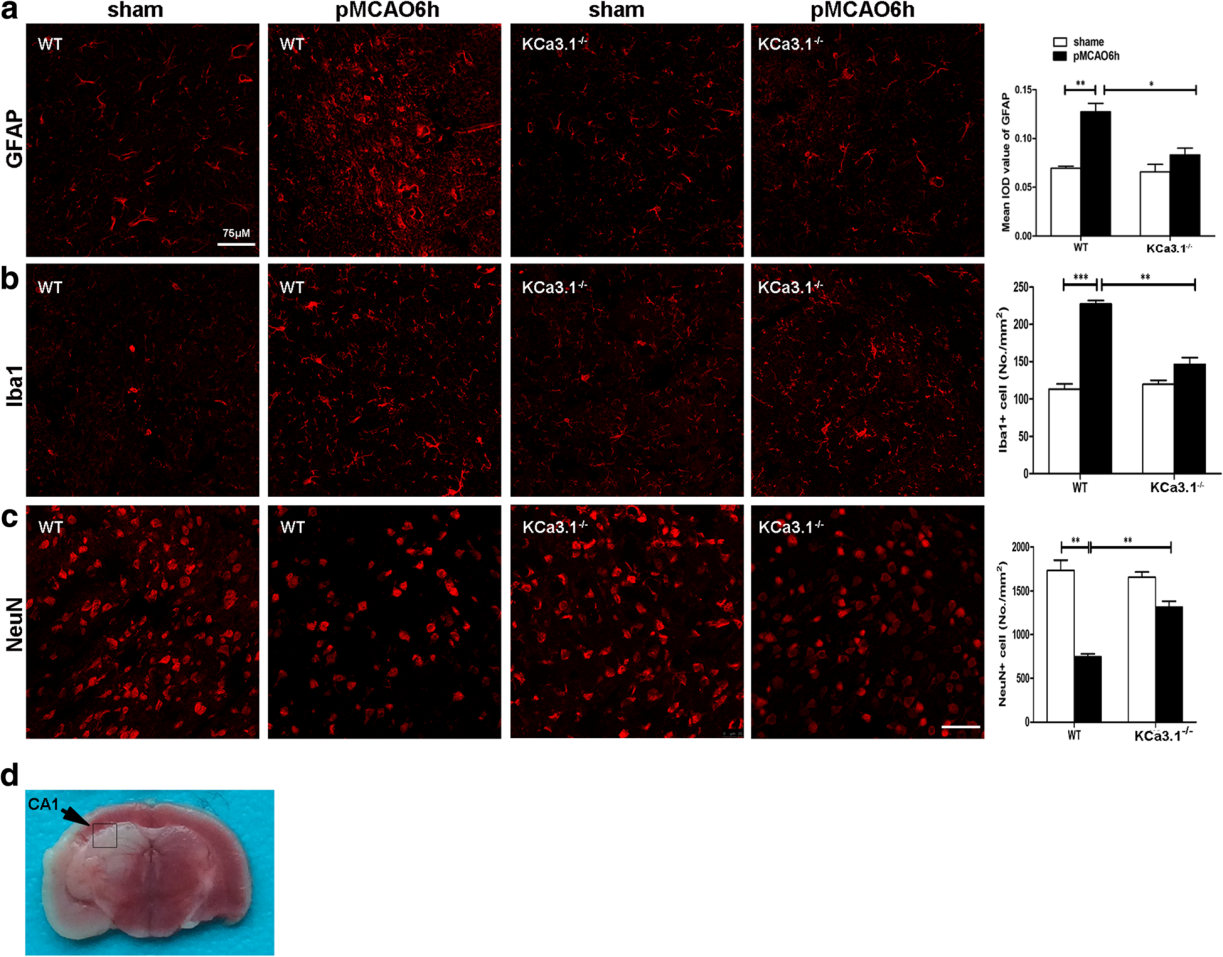
Decreased glial activation and neuronal loss in brains of KCa3.1 deletion mice following pMCAO. Reactive astrocytes (**a**), activated microglia (**b**), and neurons (**c**) from the hippocampal CA1 regions of WT or KCa3.1^−/−^ mice at 6 h after pMCAO were visualized by GFAP, Iba1, and NeuN immunostaining, respectively. At least four coronal slices from each mouse brain and at least three brains of each genotype were used for immunostaining and counting. *n* = 4 per group. Scale bar: 75 μm. Data represent means ± SEM. ^*^ *p* < 0.05, ^**^ *p* < 0.01, ^***^ *p* < 0.001, unpaired, two-tailed Student’s *t* test. **d** Images of Fig. 3a–c were taken from CA1 regions (black box). WT, wild type

It is well documented that reactive gliosis of both astrocytes and microglia are two hallmark features of ischemia and its penumbral region [28]. We previously showed in primary astrocyte cultures and an AD mouse model that inhibition of KCa3.1 attenuated the reactive glial response [7]. Furthermore, we also demonstrated that deletion or inhibition of KCa3.1 reduced astrogliosis-induced neurotoxicity in a co-culture system of neurons-astrocytes [7]. To assess the role of KCa3.1 in the process of ischemic stroke, neuron survival and glial activation were measured by immunostaining with markers of neurons, astrocyte, and microglia in WT and KCa3.1^−/−^ mice 6 h after pMCAO. Significantly fewer GFAP^+^ astrocytes (*p* < 0.05, Fig. 3a) and Iba1^+^ microglia (*p* < 0.01, Fig. 3b) were detected in the 6 h pMCAO brains of KCa3.1^−/−^ mice as compared to WT mice, while more NeuN^+^ neurons were detected in the 6 h pMCAO brains of KCa3.1^−/−^ mice as compared to WT mice (*p* < 0.01, Fig. 3c). As shown in Fig. 3d, the images of Fig. 3a–c were taken from CA1 (Additional file 3: Figure S3). Overall, these results suggest that targeted deletion of KCa3.1 has neuroprotective effects against cerebral ischemic injury induced by pMCAO.

### KCa3.1 and TRPV4 mediate oxygen-glucose deprivation induced astrogliosis

**Fig. 4:**
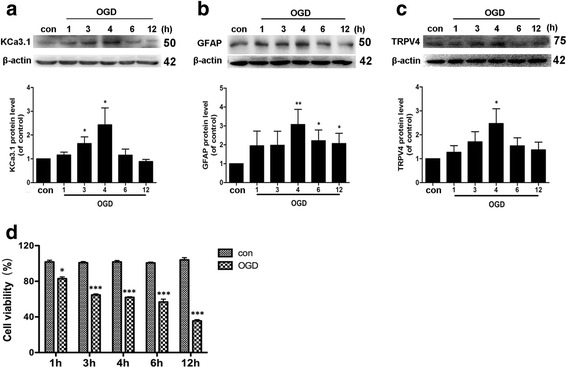
Upregulation of KCa3.1, GFAP, and TRPV4 channels following OGD in cultured astrocytes. Western blot analysis of (**a**) KCa3.1, (**b**) GFAP, and (**c**) TRPV4 expression after OGD-treatment for 0, 1, 3, 4, 6, 12 h. Data represent the means ± SEM of KCa3.1, GFAP, and TRPV4 density normalized to β-actin values for *n* = 3 cultures. ^*^ *p* < 0.05, ^**^ *p* < 0.01, one-way ANOVA followed by the Dunnett’s multiple comparison test compared with control. (**d**) Cell viability was determined by the CCK-8 assay. The values represent the percentage of cells viability induced by 1, 3, 4, 6, or 12 h of OGD (*n* = 4). Data represent means ± SEM. ^*^ *p* < 0.05, ^***^ *p* < 0.001, unpaired, two-tailed Student’s *t* test compared with control. Con control, OGD oxygen-glucose deprivation

We previously found in primary astrocyte cultures that KCa3.1 expression was increased in reactive astrocytes induced by TGF-β, while pharmacological blockade or genetic deletion of KCa3.1 attenuated astrogliosis [17]. Butenko et al. [15] reported that TRPV4 was markedly enhanced in astrocytes of the CA1 region after hypoxia/ischemia and that increased TRPV4 expression coincided with the development of astrogliosis. We therefore investigated the hypothesis that KCa3.1 regulates Ca^2+^ entry via the TRPV4 channel leading to reactive astrogliosis-induced neuronal damage during ischemia stroke. We firstly evaluated the expression of KCa3.1 and TRPV4 during the process of OGD-induced astrogliosis in vitro. Primary cultured astrocytes were subject to OGD for 1, 3, 4, 6, or 12 h to induce reactive astrogliosis as described previously [29]. As shown in Fig. 4, OGD induced a time-dependent upregulation of KCa3.1 and TRPV4 channels, which was consistent with the upregulation of GFAP (Fig. 4a–c). The cell viability analysis showed a significant decrease in astrocytes viability during 1–12 h after OGD treatment (Fig. 4d). These data suggested that the increase in KCa3.1 and TRPV4 occurred concomitantly with upregulation of GFAP during the process of OGD-induced reactive astrogliosis.

### KCa3.1 channels hyperpolarize reactive astrocytes membrane potential during oxygen-glucose deprivation

**Fig. 5:**
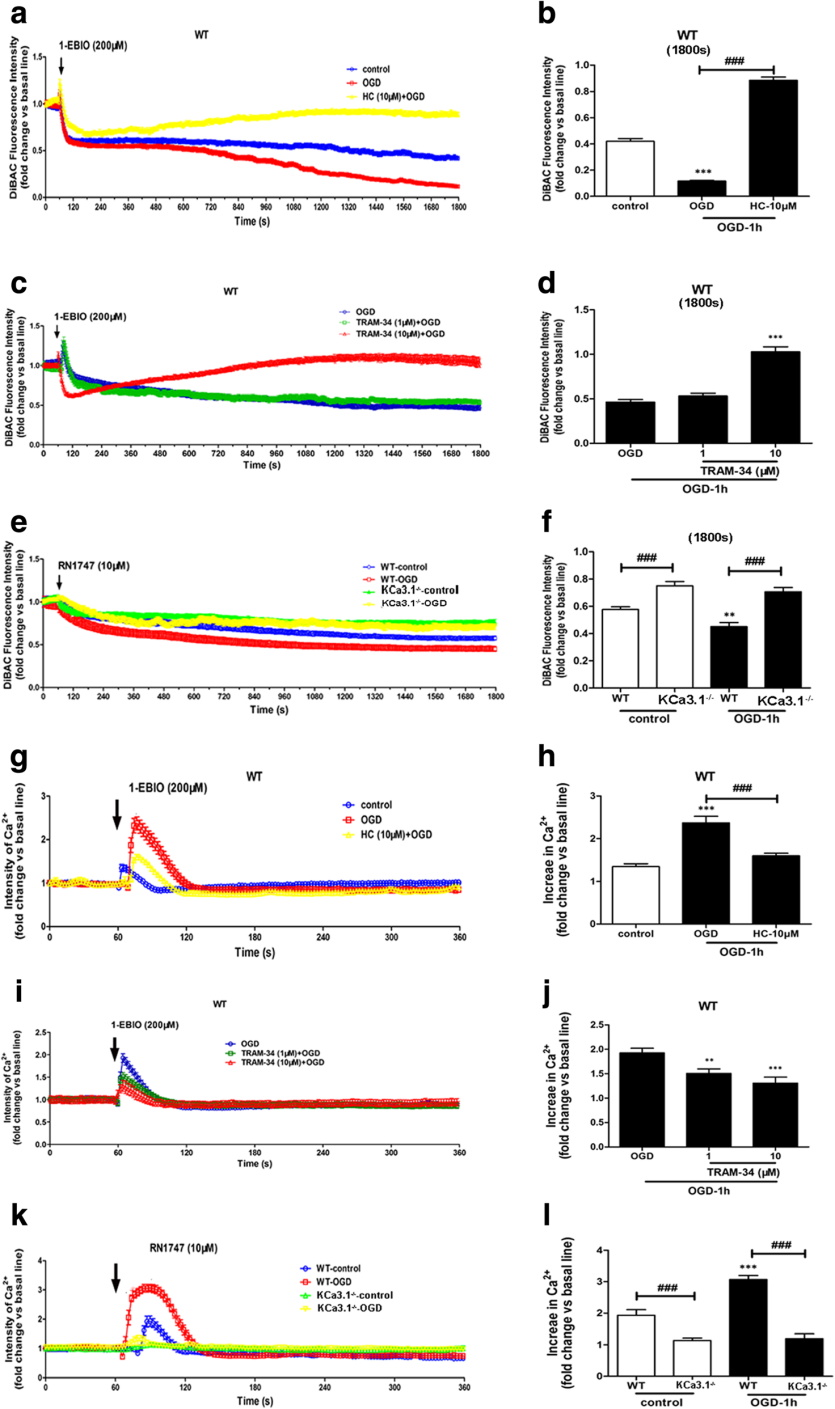
Role of TRPV4 and KCa3.1 in alteration of membrane potential and Ca^2+^ entry in astrocytes following OGD. **a–f** Changes in membrane potential in response to activation of KCa3.1 channels and TRPV4 channels in astrocytes exposed to OGD 1 h. **a**, **b** 1-EBIO was added to WT astrocytes and membrane potential measured with or without OGD or HC 067047. Data are presented as means ± SEM. *n* = 10–20. ^***^ *p* < 0.001 vs control. ^###^ *p* < 0.001 vs OGD. **c**, **d** 1-EBIO was added to WT astrocytes and membrane potential measured with or without OGD or TRAM-34 (1 and 10 μM). Data are presented as means ± SEM. *n* = 10–20. ^***^ *p* < 0.001 vs OGD. **e**, **f** RN1747 was added to WT or KCa3.1 KCa3.1^−/−^ astrocytes and membrane potential measured with or without OGD. **b**, **d**, **f** Summary of data showing the membrane potential in **a**, **c**, and e at 1800s. Data are presented as means ± SEM. *n* = 10–20. ^**^ *p* < 0.01 vs WT control, ^###^ *p* < 0.001 vs WT control or WT OGD. **g–l** Changes in [Ca^2+^]_i_ in response to activation of KCa3.1 channels and TRPV4 channels in astrocytes exposed to OGD 1 h. **g**, **h** 1-EBIO was added to WT astrocytes and [Ca^2+^]_i_ measured with or without OGD or HC 067047. Data are presented as means ± SEM. *n* = 10–20. ^***^ *p* < 0.001 vs control. ^###^ *p* < 0.001 vs OGD. **i**, **j** 1-EBIO was added to WT astrocytes and [Ca^2+^]_i_ measured with or without OGD or TRAM-34 (1 and 10 μM). Data are presented as means ± SEM. *n* = 10–20. ^**^ *p* < 0.01, ^***^ *p* < 0.001 vs OGD. **k**, **l** RN1747 was added to WT or KCa3.1 KCa3.1^−/−^ astrocytes and [Ca^2+^]_i_ measured with or without OGD. **h**, **j**, **l** Summary of data showing the [Ca^2+^]_i_ in **h**, **j**, and **l** at 70–80 s. Data are presented as means ± SEM. *n* = 10–20. ^***^ *p* < 0.001 vs WT control, ^###^ *p* < 0.001 vs WT control or WT OGD. Statistical analysis was performed using unpaired, two-tailed Student’s *t* test. 1-EBIO, 1-ethyl-2-benzimidazolinone, OGD oxygen-glucose deprivation, WT wild type, HC HC 067047

To further establish the relationship between KCa3.1 and TRPV4 in regulating reactive astrocytes during ischemia stroke, we investigated the role of TRPV4 in the process of KCa3.1 regulated membrane potential. As reported before, activation of KCa3.1 hyperpolarizes non-excitable cells such as airway smooth muscle cells [25] and pancreatic cancer cells [30]; this hyperpolarization enhances the driving force for Ca^2+^ influx. To evaluate the role of KCa3.1 in OGD activation of astrocytes, the KCa3.1 pharmacological activator 1-ethylbenzimidazolinone (EBIO) was used to activate KCa3.1 [31] with and without OGD. As shown in Fig. 5a, 200 μM 1-EBIO induced a larger hyperpolarization (as measured by DiBAC4(3) fluorescence intensity) in the 1 h OGD-treated astrocytes as compared to control cells, likely due to enhanced K^+^ efflux upon increased KCa3.1 activation. We then used the TRPV4 channel antagonist HC 067047 to test the role of TRPV4 channels in regulating membrane potential during 1-EBIO-induced KCa3.1 activation. Blockade of TRPV4 attenuated the level of 1-EBIO-mediated membrane hyperpolarization in OGD-induced astrocytes (*p* < 0.001, Fig. 5a, b). In addition, 10 μM, but not 1 μM, KCa3.1 selective blocker TRAM-34 attenuated 200 μM 1-EBIO-induced membrane hyperpolarization in 1 h OGD-treated astrocytes (*p* < 0.001, Fig. 5c, d).

It was reported that as the downstream component of the TRPV4 transduction pathway, a genetic deficit of KCa3.1 reduced lung damage and pulmonary circulatory collapse induced by TRPV4 channel activation [32]. We therefore measured the change in membrane potential in both WT and KCa3.1 KCa3.1^−/−^ astrocytes following the addition of the TRPV4 channel agonist 10 μM RN1747 along with OGD. We found that hyperpolarization in response to RN1747 was reduced in the KCa3.1^−/−^ astrocytes, compared with the WT cells (*p* < 0.001, Fig. 5e, f), both in normoxia and in response to OGD.

### KCa3.1 activation induces calcium entry in astrocytes through TRPV4 calcium channel during oxygen-glucose deprivation

It has been suggested that the Ca^2+^ influx resulting from activation of TRPV4 channels is leveraged by the high Ca^2+^ sensitivity of KCa3.1 and KCa2 to cause vascular endothelium vasodilation [33]. To evaluate the potential activation of KCa3.1 channels by OGD, we measured [Ca^2+^]_i_ in response to activation of KCa3.1 channels by 1-EBIO in control astrocytes and in astrocytes subjected to 1 h of OGD. We found that 200 μM 1-EBIO induced a larger increase in [Ca^2+^]_i_ in the 1 h OGD-treated astrocytes as compared to the control cells (*p* < 0.001, Fig. 5g, h). We then used HC 067047 to test the role of TRPV4 channels in the Ca^2+^ influx during OGD and 1-EBIO-induced KCa3.1 activation; we found that blockade of TRPV4 channels attenuated the level of 1-EBIO-mediated Ca^2+^ increase in OGD-induced astrocytes (*p* < 0.001, Fig. 5g, h). To further confirm that KCa3.1 activation induced the [Ca^2+^]_i_ increase, TRAM-34 (1 and 10 μM) was used in the same experiments. In the presence of 1 and 10 μM TRAM-34, the [Ca^2+^]_i_ increase induced by 200 μM 1-EBIO was attenuated in both the 1 μM (*p* < 0.01, Fig. 5i, j) and 10 μM (*p* < 0.001, Fig. 5i, j) TRAM-34 treatment of 1 h OGD-astrocytes.

To evaluate the effect of OGD on TRPV4 channels, we used 10 μM RN1747 in WT and KCa3.1^−/−^ astrocytes with and without OGD. [Ca^2+^]_i_ was measured before and after the application of OGD 1 h. In the KCa3.1^−/−^ astrocytes, the Ca^2+^ influx induced by RN1747 was drastically reduced as compared with the WT cells (*p* < 0.001, Fig. 5k, l). Taken together, our data showed that TRPV4 channels mediated the plasma membrane hyperpolarization-evoked [Ca^2+^]_i_ increase due to activation of KCa3.1.

### KCa3.1 and TRPV4 channels regulated astrogliosis

**Fig. 6:**
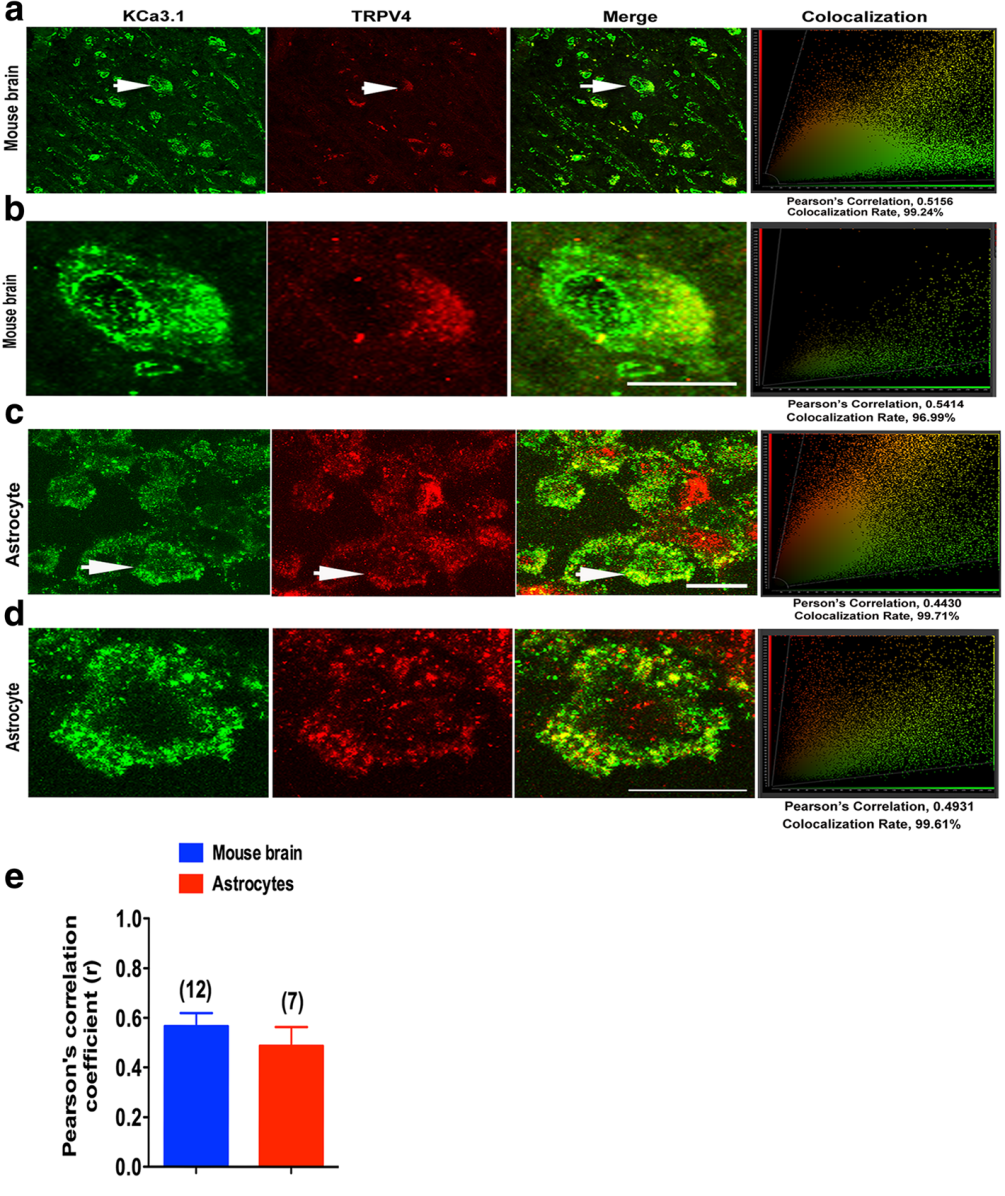
KCa3.1 and TRPV4 co-localized in primary cultured astrocytes and mouse brain cortex. Double immunofluorescence images of KCa3.1 (green) and TRPV4 (red) in normal mouse brains (**a**, **b**), and primary cultured astrocytes (**c**, **d**). Note the strong co-localization indicated by merge yellow fluorescence, quantification of the co-localization observed in experiments as shown in g. (**e**) The histograms represent the ratio of the mean Pearson correlation coefficient calculated from the co-labeling in a number of samples, as indicated above the bar. Scale bar: 25 μm

The above results indicate that KCa3.1 and TRPV4 are functionally linked. The question then arises as to whether they are physically associated with each other. The physical interaction between KCa3.1 and TRPV4 channels was further tested. Confocal analysis of double-labeled staining showed that KCa3.1 and TRPV4 channels co-localized in normal mouse brain (Fig. 6a, b) and primary cultured astrocytes (Fig. 6c, d). Histograms represent the ratio of the mean Pearson correlation coefficient using Leica LAS AF Lite software. The Pearson correlation values range from − 1 to + 1. A correlation of 1 indicates complete co-localization between the two proteins. A correlation of − 1 indicates a negative interaction, and a correlation of 0 indicates no co-localization between the two proteins. When double-labeled staining of KCa3.1 and TRPV4 channels, co-localization was observed in normal mouse brain (Fig. 6a, b) and primary cultured astrocytes (Fig. 6c, d). As shown in Fig. 6e, Pearson correlation coefficients are 0.57 (mouse brain) and 0.49 (astrocytes), confirming co-localization between KCa3.1 and TRPV4.

**Fig. 7:**
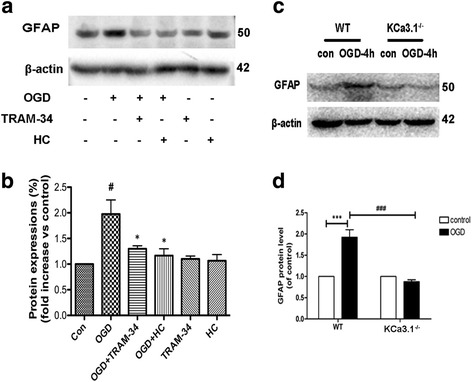
Involvement of KCa3.1 in OGD-induced reactive astrogliosis. **a**, **b** Representative western blot showing GFAP expression in cultured astrocytes treated with OGD for 4 h in the presence of 1 μM TRAM-34 and 10 μM HC 067047. Quantification of western blot for GFAP expression (*n* = 3). Data are presented as means ± SEM. ^#^ *p* < 0.05 vs control, ^*^ *p* < 0.05 vs OGD alone. **c**, **d** Representative western blot and quantification of GFAP expression after 4 h OGD treatment in WT and KCa3.1 KCa3.1^−/−^ astrocytes (*n* = 3–4). ^***^ *p* < 0.001, ^###^ *p* < 0.001. One-way ANOVA followed by the Dunnett’s multiple comparison test. Con control, WT wild type

The involvement of the KCa3.1 and TRPV4 in regulating the emergence of astrogliosis induced by 4 h OGD was studied by using antagonists of these two channels. In the presence of either TRAM-34 (1 μM) or HC 067047 (10 μM), upregulation of GFAP was attenuated (*p* < 0.05, Fig. 7a, b). Moreover, astrocytes from KCa3.1 KCa3.1^−/−^ mice exhibited a decrease in GFAP expression induced by 4 h OGD treatment (*p* < 0.001, Fig. 7c, d) as compared to WT astrocytes. These results suggested that KCa3.1 and TRPV4 are both involved in the process of astrogliosis induced by OGD.

## Discussion

The major findings of this study are that the KCa3.1 channel contributes to reactive astrogliosis following ischemia, as shown in both OGD-treated astrocytes in vitro and the brains of mice subject to pMCAO in vivo, that KCa3.1 regulated Ca^2+^ influx and membrane potential by functional cooperation with the TRPV4 channel in OGD-treated astrocytes. The expression of both channels was increased during astrogliosis and blockade either KCa3.1 or TRPV4 attenuated the astrogliosis process. Additionally, double-labeled staining experiments showed the co-localization between TRPV4 and KCa3.1 in mouse brain and primary astrocytes, where they might cooperate to regulate the reactive astrogliosis.

We provided genetic target validation of the role of KCa3.1 by demonstrating that mice with a gene deletion of KCa3.1 exhibited significantly reduced pathology after pMCAO. This was manifested as both reduced infarct area after the insult as well as reduced astrogliosis, microglial activation and neuron loss. This is consistent with recent studies that demonstrated that pharmacological blockade of KCa3.1 or gene silence reduces infarct size and other neurological deficits in rats or mice [34]. Moreover, in the present study, we extended this hypothesis to show that KCa3.1 acted as an endogenous sensor of Ca^2+^ influx caused by activation of TRPV4. We found that blockade of either channel could reduce astrogliosis. In addition, we found that these channels could be co-localization.

The lack of voltage-gated Ca^2+^ channels in non-excitable cells allows KCa3.1 to act as Ca^2+^ detectors and Ca^2+^ amplifiers. Activation of KCa3.1 induced K^+^ efflux and membrane hyperpolarization, which ultimately upregulated the driving force for Ca^2+^ influx [35, 36], and further KCa3.1 channels will be activated in a positive feedback loop. KCa3.1-Ca^2+^ channels were involved in non-excitable cells activation process [35]. An interaction between KCa3.1 and TRPV4 channels has been found in several diseases, during which Ca^2+^ dynamics induced by KCa3.1 were dependent on Ca^2+^ influx via TRPV4 channels [37, 38]. There was a functional co7upling between KCa3.1 and TRPV4 to regulate Ca^2+^ levels leading to pulmonary circulatory collapse and hemorrhage [39].

An interaction of TRPV4 and KCa channels has been found in several other brain functions. In retinal ganglion cells, activation of TRPV4 induced apoptosis due to Ca^2+^ overload [40]. Shi et al. [41] reported that Ca^2+^ influx via TRPV4 channels involved in infrasound-induced activation of astrocytes and microglia, and then neuronal death. Astrocytes volume was regulated by TRPV4/AQP4 complex through water transport and calcium homeostasis. Over activation of the interaction of TRPV4/AQP4 complex would trigger the pathological swelling and reactive gliosis [42]. Ca^2+^ influx through TRPV4 channels activated the large Ca^2+^-activated K^+^ channel (BK), and TRPV4/BK functional coupling regulated bladder contractility in the storage phase [43]. Gene deletion of KCa3.1 channels attenuated lung damage and pulmonary circulatory collapse caused by TRPV4 activation. In addition, these data are consistent with recent studies that suggested that the KCa channels might serve as amplifiers of the Ca^2+^ signaling through TRP channels [44] and data demonstrating a coupling of the two types of channels in osmosensors in the paraventricular nucleus [45]. Recently, it was reported that calcium-gated K^+^ channels of the KCa1.1- and KCa3.1-type couple intracellular Ca^2+^ signals to membrane hyperpolarization in mesenchymal stromal cells from the human adipose tissue (Tarasov et al. 2017). It might explain the reason that stimulation of TRPV4 agonist RN1747 still caused membrane hyperpolarization in the KCa3.1^−/−^ astrocytes as shown in Fig. 6e.

Reactive astrocytes are involved in many pathological processes of CNS diseases, such as stroke, traumatic brain injury, and AD [46]. During the process of neuroinflammation, activated microglia induce the phenotypic switch of astrocytes from a quiescent to a reactive phenotype. Neurotoxic reactive astrocytes, termed A1 astrocytes, are induced by activated microglia, which lose the ability to support neurons and oligodendrocyte survival and synaptogenesis [47]. In our previous studies, we showed that a similar phenotype can be induced in culture using TGF-β [17], and that blockade or deletion of the KCa3.1 channel prevented the emergence of the reactive phenotype. Whether this mechanism of astrocyte activation depends upon the astrocytic TRPV4 channels is a subject for future investigation.

KCa3.1 is thought to regulate microglial activation such as migration and neurotoxicity induced by activated microglia in vitro [48, 49]. Higher densities of Kv1.3, KCa3.1, and Kir2.1 currents were detected in microglia from the infarcted area of reversible MCAO than that from non-infarcted control brains. Similarly, strong KCa3.1 immunoreactivity was also found on activated microglia/macrophages of human infarcts [34]. However, we did not find any obvious co-localization between KCa3.1 and microglia in the brains of pMCAO mice by immunofluorescence staining. It may be that the different model of ischemic stroke (reversible MCAO vs pMCAO) as well as the different detection method (patch clamp vs immunofluorescence staining) might be the reason.

## Conclusions

In conclusion, these data suggested that KCa3.1 and TRPV4 channels forms a signaling complex involved in maintaining abnormally high [Ca^2+^]_i_ levels to drive phenotype switch of astrogliosis during ischemia stroke. Gene deletion of KCa3.1 attenuated infarct area and reduced neuron loss and gliosis in pMCAO, which may have therapeutic utility in ischemia stroke associated with abnormally high [Ca^2+^]_i_ in astrogliosis.

## Additional files

Additional file 1: **Figure S1.** Cerebral blood flow (CBF) of ischemic brain hemisphere before and during permanent middle cerebral artery occlusion (pMCAO) was monitored by transcranial laser Doppler. The arrow depicted the start of pMCAO. (TIFF 58 kb)

Additional file 2: **Figure S2.** Western blot analysis of lysates from 10-week-old male WT mice following 1, 3, 6, or 12 h of pMCAO analyzed by antibodies to KCa3.1 (A) and GFAP (B). (TIFF 223 kb)

Additional file 3: **Figure S3.** GFAP^+^ reactive astrocytes and Iba1^+^ activated microglia from the cortex and hippocampal regions of WT or KCa3.1^−/−^ mice brain at 6 h after pMCAO were visualized by immunostaining. Scale bar: 200 μm. WT, wild type. (TIFF 7211 kb)

## Abbreviations

1-EBIO: 1-Ethylbenzimidazolinone
GFAP: Glial fibrillary acidic protein
KCa3.1: Intermediate-conductance calcium-activated potassium channel
pMCAO: Permanent middle cerebral artery occlusion
TRAM-34: 1-((2-chlorophenyl) (diphenyl) methyl)-1H–pyrazole
TRPV4: Transient receptor potential vanilloid 4
