# Supplementary figures and images for: The potassium channel KCa3.1 constitutes a pharmacological target for astrogliosis associated with ischemia stroke
Source: J Neuroinflammation. 2017 Oct 16;14:203. doi: 10.1186/s12974-017-0973-8 (PMC5644250; doi:10.1186/s12974-017-0973-8)

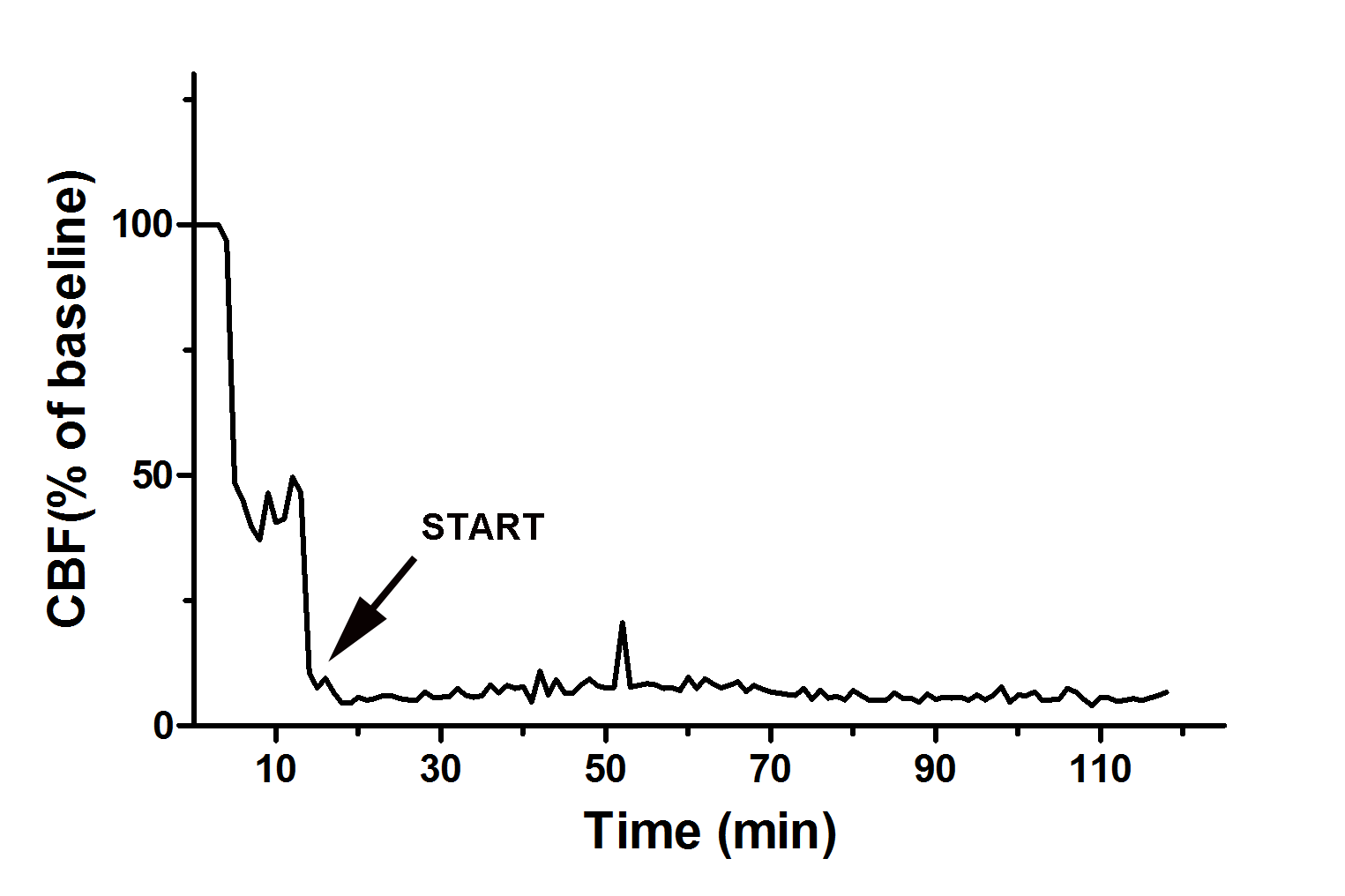

Supplement: Supplementary file 1 — Figure S1. Cerebral blood flow (CBF) of ischemic brain hemisphere before and during permanent middle cerebral artery occlusion (pMCAO) was monitored by transcranial laser Doppler. The arrow depicted the start of pMCAO. (TIFF 58 kb) [file 12974_2017_973_MOESM1_ESM.tif]

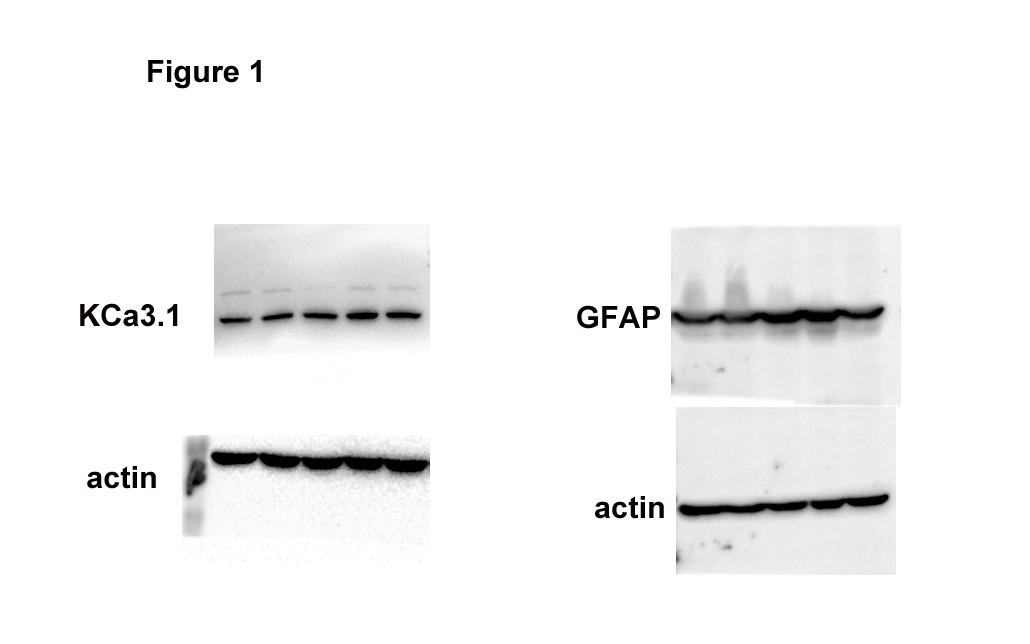

Supplement: Supplementary file 2 — Figure S2. Western blot analysis of lysates from 10-week-old male WT mice following 1, 3, 6, or 12 h of pMCAO analyzed by antibodies to KCa3.1 (A) and GFAP (B). (TIFF 223 kb) [file 12974_2017_973_MOESM2_ESM.tif]

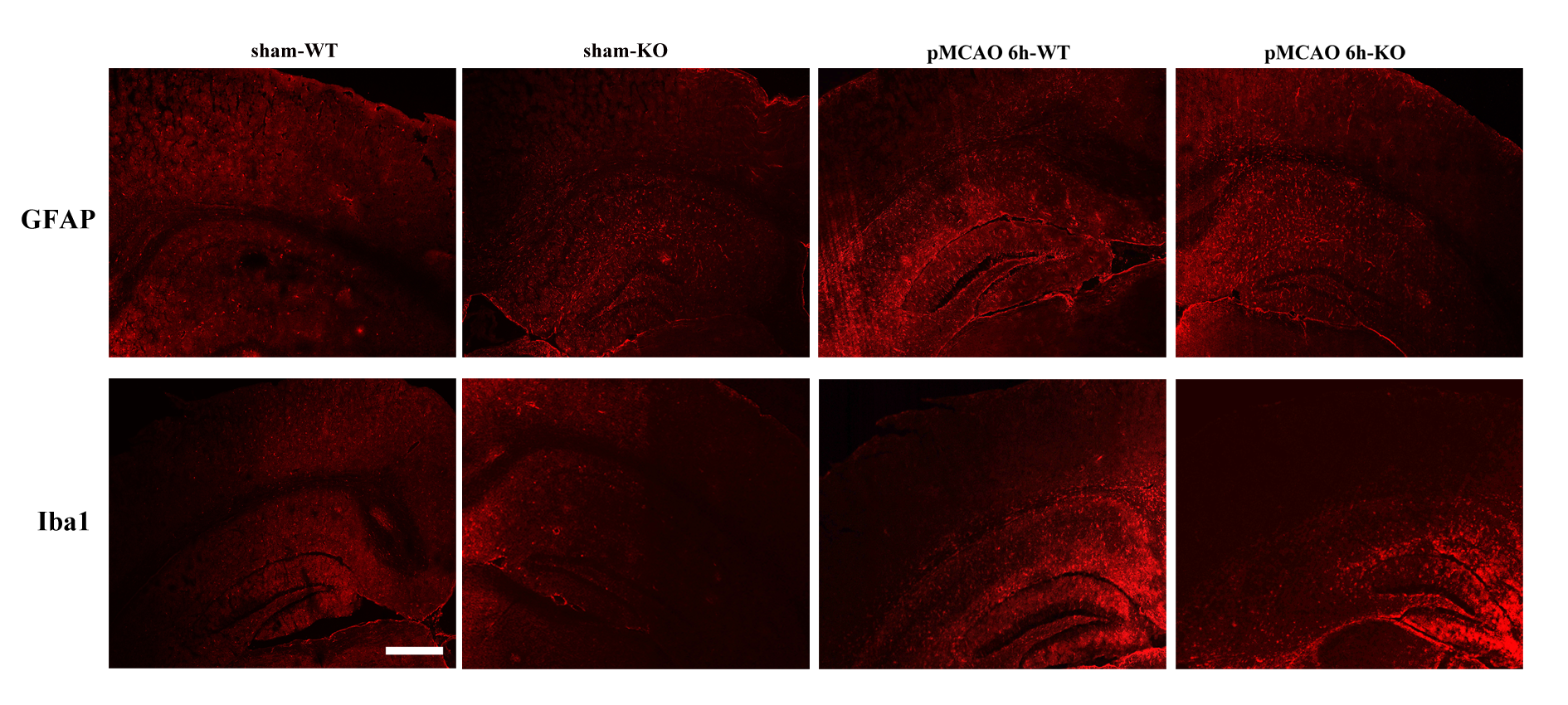

Supplement: Supplementary file 3 — Figure S3. GFAP+ reactive astrocytes and Iba1+ activated microglia from the cortex and hippocampal regions of WT or KCa3.1−/− mice brain at 6 h after pMCAO were visualized by immunostaining. Scale bar: 200 μm. WT, wild type. (TIFF 7211 kb) [file 12974_2017_973_MOESM3_ESM.tif]
